# Activated α_2_-macroglobulin binding to cell surface GRP78 induces trophoblastic cell fusion

**DOI:** 10.1038/s41598-020-66554-0

**Published:** 2020-06-15

**Authors:** Daniel Bastida-Ruiz, Christine Wuillemin, Aude Pederencino, Michal Yaron, Begoña Martinez de Tejada, Salvatore Vincent Pizzo, Marie Cohen

**Affiliations:** 1 Department of Pediatrics, Gynecology and Obstetrics, Faculty of Medicine, University of Geneva, 1206 Geneva, Switzerland; 2 Department of Pathology, Duke University Medical Center, Durham, North Carolina 27710 USA

**Keywords:** Cell biology, Developmental biology

## Abstract

The villous cytotrophoblastic cells have the ability to fuse and differentiate, forming the syncytiotrophoblast (STB). The syncytialisation process is essential for placentation. Nevertheless, the mechanisms involved in cell fusion and differentiation are yet to be fully elucidated. It has been suggested that cell surface glucose-regulated protein 78 (GRP78) was involved in this process. In multiple cancer cells, cell membrane-located GRP78 has been reported to act as a receptor binding to the active form of α_2_-macroglobulin (α_2_M*), activating thus several cellular signalling pathways implicated in cell growth and survival. We hypothesised that GRP78 interaction with α_2_M* may also activate signalling pathways in trophoblastic cells, which, in turn, may promote cell fusion. Here, we observed that α_2_M mRNA is highly expressed in trophoblastic cells, whereas it is not expressed in the choriocarcinoma cell line BeWo. We thus took advantage of forskolin-induced syncytialisation of BeWo cells to study the effect of exogenous α_2_M* on syncytialisation. We first demonstrated that α_2_M* induced trophoblastic cell fusion. This effect is dependent on α_2_M*-GRP78 interaction, ERK1/2 and CREB phosphorylation, and unfolded protein response (UPR) activation. Overall, these data provide novel insights into the signalling molecules and mechanisms regulating trophoblastic cell fusion.

## Introduction

Fetal development is a complex process taking place during pregnancy with the objective of generating a viable entity^1^. The first step in this process is fertilisation, the fusion of the egg and sperm, which leads to the formation of a zygote^2^. The zygote contains all the genetic information necessary to form an individual and the extra-fetal structures required for fetal development such as the placenta^3^.

The placenta is the transient organ in charge of nourishing, oxygenising and protecting the developing fetus^4^. Its unique conformation allows for the exchange of molecules necessary for fetal development by linking the fetal and maternal circulations^5^. In particular, these interchanges are performed through the chorionic villi, highly vascularised structures that sprout from the chorion to provide a maximal contact surface with maternal blood in the intervillous space^6,7^. The surface of the chorionic villi is formed by a continuous multinucleated monolayer referred to as syncytiotrophoblast (STB)^8^. The STB is the structure in charge of the exchange of nutrients and gas and acts as an immunological barrier and hormone secretion organ^6^. Unconventional STB formation has been the subject of study for decades, and it is now well-established that its origin lies in cell fusion^9^. In fact, under the STB reside the mononucleated villous cytotrophoblastic cells (vCTB), which participate in STB formation and cellular turnover by asymmetric division^9^. During vCTB division, a proliferative daughter cell that will continue dividing, together with a second daughter cell that will fuse and differentiate into the STB, are generated^10^. The vCTB cell fusion and differentiation process that gives rise to STB is called syncytialisation, and it has been reported to be essential for correct placentation^11^. Specifically, an acceleration of the fusion process may drain the regenerative pool of vCTB, whereas insufficient cell fusion may lead to an abnormal STB layer and subsequent functional deficits^12^. For this reason, understanding the mechanisms involved in syncytialisation is essential to prevent aberrant placenta formation.

Several factors implicated in syncytialisation have been identified, which include the expression of fusogenic proteins such as syncytin-1, syncytin-2 and their receptors^12,13^. Caspase 8 activation, allowing phosphatidylserine flip and cytoskeleton rearrangement of vCTB, together with cell cycle cessation, have also been described as syncytialisation factors^14,15^. Recently, we identified the unfolding protein response (UPR) as an autophagy regulator in trophoblastic cells during syncytialisation^16^. The UPR is an adaptive mechanism activated under cellular stress situations, which is triggered by the glucose-regulated protein 78 (GRP78), an endoplasmic reticulum (ER) resident protein belonging to the heat shock protein 70 (HSP70) family^17^. Interestingly, GRP78 has been observed at the cell surface of trophoblastic cells where it may play a role in syncytialisation^18^. Indeed, it was described that decreased expression of GRP78 by siRNA treatment or by blocking membrane GRP78 with C20 anti-GRP78 antibodies diminished trophoblastic cell fusion capacities *in vitro*^19^. Additionally, decreased expression of GRP78 at the cell surface of trophoblastic cells due to an impaired relocation mechanism^19^ was observed in women who developed preeclampsia (PE), a maternal syndrome characterised by abnormal placentation^20^. Altogether, this information suggests cell surface-located GRP78 plays a role in trophoblastic syncytialisation.

**Figure 1:**
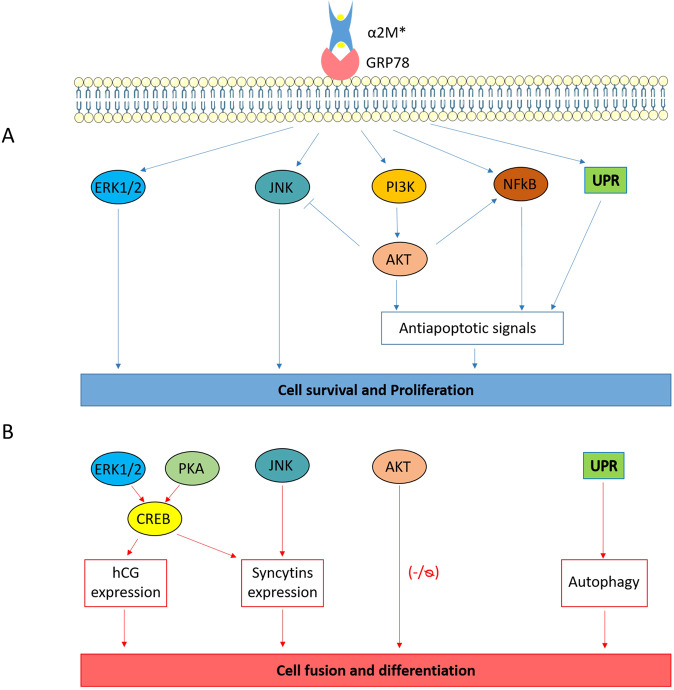
Schematic representation of signalling cascades involved in the cell survival of prostate cancer cells stimulated by α_2_M-GRP78 interaction and the role of these signalling cascades in syncytialisation. (**A**) α_2_M-GRP78 interaction in prostate cancer cells. Interaction between GRP78 and α_2_M* promotes the activation of ERK1/2, JNK, PI3K, Akt, NF-kB and UPR in prostate cancer cells. The activation of ERK1/2 and JNK is known to enhance cell survival and proliferation through their derived signalling pathways. Additionally, the activation of Akt, NF-kB and UPR is known to promote anti-apoptotic signals, which, in turn, promote cell survival and proliferation. Overall, the proteins activated by α_2_M-GRP78 interaction have been reported to enhance the survival and proliferation of prostate cancer cells^22,29^. (**B**) Effects of ERK1/2, PKA, CREB, JNK, Akt signaling pathways and UPR activation on syncytialisation. The activated forms of ERK1/2 and PKA are known to activate CREB, a protein that has been reported to increase the expression of hCG and syncytins, promoting trophoblastic cell fusion and differentiation^30^. Furthermore, active JNK has been shown as well to increase the expression of syncytins^31^. In a more controversial manner, Akt activation was described as a negative modulator of syncytialisation in trophoblastic cells^32^. However, some studies confer no effects on cell fusion and differentiation to this protein^30^. Finally, UPR was recently reported to activate autophagy during trophoblastic syncytialisation, promoting cell survival and favouring cell fusion and differentiation^16^. Summing up, some of the proteins activated in prostate cancer cells by GRP78-α_2_M* interaction are capable of inducing syncytialisation in trophoblastic cells.

The presence of GRP78 at the cell surface has also been observed in a wide variety of cancer cells such as ovarian^21^, prostate^22^, breast^23^, gastric^24^ and pancreatic^25^ cancer cells. Research on the potential role of membrane GRP78 in cancer cells is more advanced than in vCTB, and several signalling pathways induced by the binding of proteins to cell surface GRP78 have been identified [review^26^]. One of the most studied GRP78 binding partners is the α_2_-macroglobulin (α_2_M), an abundant plasma protein and a primary plasma proteinase inhibitor^27^. This glycoprotein is a homo-tetramer with 180-KDa subunits, which needs to be modified by proteinases to generate its final active form, α_2_M*^28^. In prostate cancer cells, the binding of α_2_M* to cell surface GRP78 triggers several signalling pathways related to cell survival and proliferation^22,29^ (Fig. 1A). Extracellular signal-regulated protein kinases 1 and 2 (ERK1/2) and c-Jun N-terminal kinase (JNK) are activated by α_2_M*-GRP78 interaction and lead to cell proliferation induction^22^. Additionally, the interaction of GRP78 and α_2_M* promotes the Ras and Phosphatidylinositol-4,5-bisphosphate 3-kinase (PI3K)-dependent signalling pathway, triggering the Protein Kinase B (Akt)^22,29^ and nuclear factor kappa-light-chain-enhancer of activated B cells (NF-kB), which enhance cell survival^29^. Finally, it was observed that in prostate cancer, GRP78-α_2_M* interaction induces UPR by increasing GRP78, eukaryotic translation initiation factor 2α (eIF2α), activating transcription factor (ATF)4 and ATF6 expression^29^.

Interestingly, some of these pathways were previously reported to be implicated in cell fusion and differentiation (Fig. 1B). For instance, in trophoblastic cells, ERK1/2 and protein kinase A (PKA) are capable of activating the cAMP response element-binding protein (CREB), which, in turn, increases the expression of syncytins and human chorionic gonadotropin (hCG), a hormone secreted by STB that promotes cell fusion^30^. JNK is also involved in the expression of syncytins, promoting cell fusion^31^. On the contrary, evidence of Akt involvement in trophoblastic syncytialisation is more inconsistent, with some studies suggesting a negative effect on syncytialisation^32^, while others conferred no cell fusion and differentiation effects at all to Akt^30^. Moreover, we recently described UPR as an adaptive mechanism necessary for correct syncytialisation through the activation of autophagy in trophoblastic cells^16^. Altogether, deciphering the possible effects of GRP78 and α_2_M* interaction on trophoblastic cells may be pertinent to better understand the syncytialisation process.

## Results

### High α_2_M expression in vCTB prevents the use of these cells in studying the effects of α_2_M on syncytialisation

**Figure 2:**
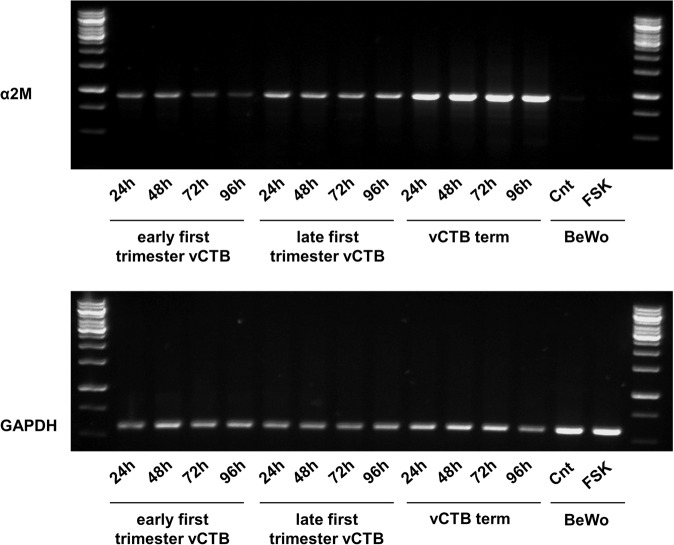
α_2_M expression in primary villous cytotrophoblastic cells and BeWo cells. vCTB were purified from early (8 weeks of gestation) and late first-trimester (11 weeks of gestation) trophoblast and term placenta and seeded for 24, 48, 72 and 96 h. BeWo cells were seeded and treated or not with 20 µM Forskolin (FSK) for 48 h. RNA was retrotranscribed, and 50 ng of cDNA was used to perform α_2_M and GAPDH PCR. n = 3.

In order to determine the best model to study the effects of α_2_M* in trophoblastic cell fusion, we first evaluated the mRNA expression of α_2_M in placental vCTB and in BeWo cells, which are commonly used as a model to mimic the syncytialisation of placental villous cytotrophoblast. We cultured vCTB cells purified from early first-trimester placenta, late first-trimester placenta and term placenta for 24 h, 48 h, 72 h and 96 h to obtain a time course of syncytialisation (as previously described by Bastida-Ruiz *et al*.^16^), allowing us to analyse basal α_2_M expression and determine if cell fusion increases the expression of α_2_M in trophoblastic cells. With a similar objective, BeWo cells were cultured for 48 h with or without Forskolin (FSK), a cell fusion inducer. mRNA expression of α_2_M was detected in primary trophoblastic cells observing that the α_2_M expression was independent of cell fusion rate, though it increased with gestational age (Fig. 2). On the contrary, no α_2_M expression was detected in treated or untreated BeWo cells (Fig. 2). The technical difficulties we encountered while trying to silence α_2_M expression in primary vCTB cells due to a low transfection efficiency led us to use BeWo cells to study the effects of exogenous α_2_M* on trophoblastic cell fusion and differentiation.

### α_2_M* increases cell fusion through its interaction with cell membrane-located GRP78

**Figure 3:**
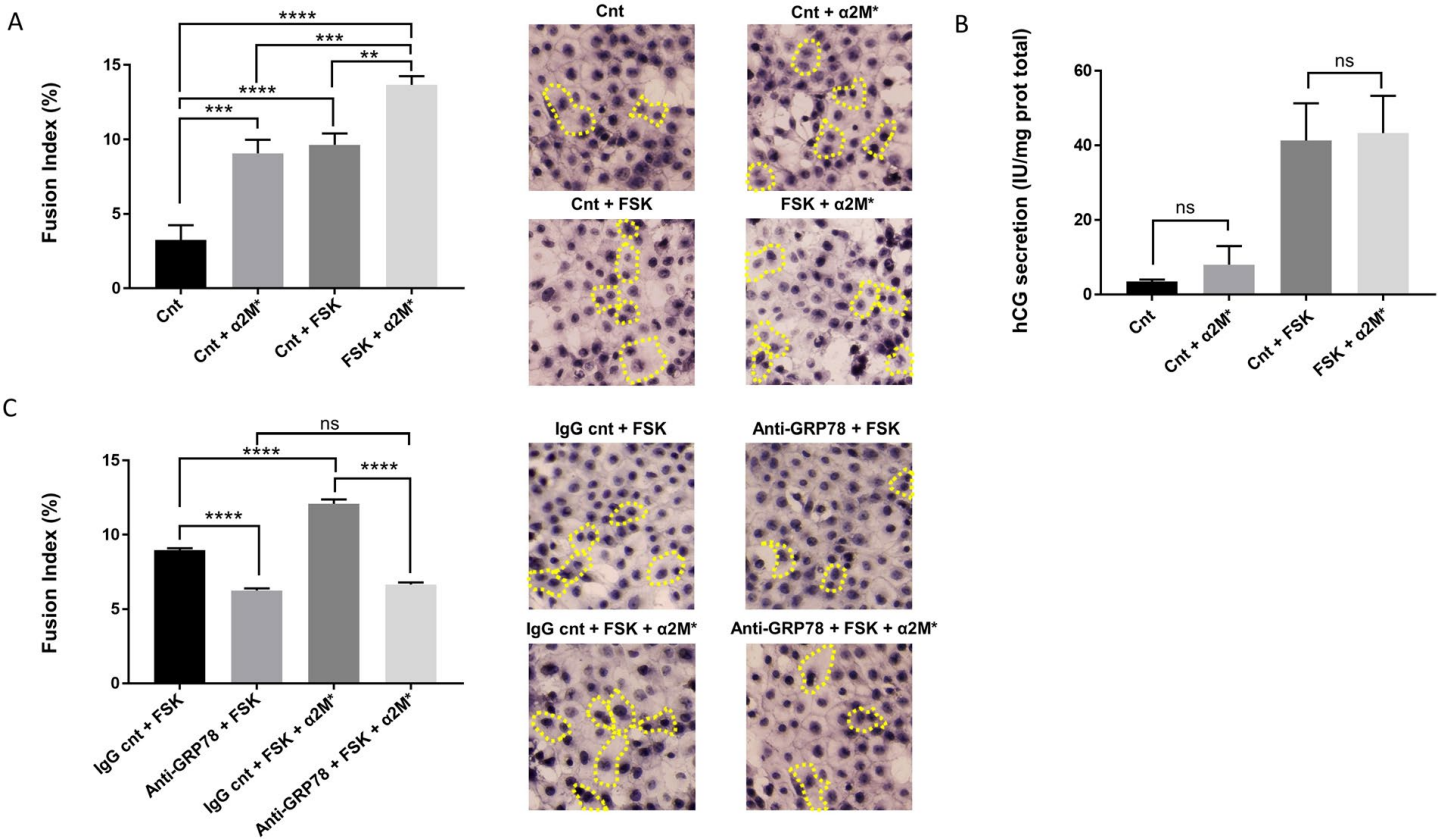
α_2_M* induces cell fusion through membrane GRP78 interaction. a-b. BeWo cells were seeded for 24 h prior to treatment with or without 20 µM Forskolin (FSK) in the presence or not of 100 pM of α_2_M* for 48 h. (**A**) Nuclei and syncytia were counted, and a fusion index was calculated. *n* = 3. Data represented as mean±SEM. ns (not significant), **P ≤ 0.01, ***P ≤ 0.005, ****P ≤ 0.001; ANOVA comparison test. The cell membrane was stained with anti-desmoplakin antibodies and nuclei with haematoxylin prior to visualisation under inverted microscopy. Syncytia delineated in yellow. (**B**) hCG levels were quantified and normalised to the corresponding total protein content. *n* = 3. Data represented as mean±SEM. ns (not significant); t-test comparison test **c**. BeWo cells were seeded for 24 h prior to treatment with 20 µM Forskolin (FSK), 100 pM of α_2_M* and 3 µg/mL of anti-GRP78 antibody or isotypic control rabbit IgG antibody for 48 h. Nuclei and syncytia were counted, and a fusion index was calculated. *n* = 3. Data represented as mean±SEM. ns (not significant), **P ≤ 0.01, ***P ≤ 0.005; ANOVA comparison test. The cell membrane was stained with anti-desmoplakin antibodies and nuclei stained with haematoxylin prior to visualisation under inverted microscopy. Syncytia delineated in yellow.

We hypothesised that α_2_M* binding to cell membrane-located GRP78 would activate signalling pathways that, in turn, may increase syncytialisation in trophoblastic cells. Firstly, we measured the fusion index (FI), a calculation that indicates the fusion rate in BeWo cells after α_2_M* treatment. We observed an increased cell fusion rate upon α_2_M* addition, which was independent of FSK treatment (Fig. 3A). Nevertheless, α_2_M* treatment exhibited no effect on hCG expression (Fig. 3B), demonstrating that α2M* induces trophoblastic cell fusion but not cell differentiation. We then decided to determine if the increased cell fusion that we observed after α_2_M* treatment was caused by its interaction with cell membrane GRP78. The blockage of GRP78 with anti-GRP78 antibodies prior to treatment of BeWo cells *in vitro* with α_2_M* caused a decrease in cell fusion, reaching the levels of α_2_M*-untreated BeWo cells (Fig. 3C). These results collectively demonstrated that cell fusion events are favoured by the interaction of α_2_M* and cell membrane GRP78 in BeWo cells.

### α_2_M*-GRP78 interaction increases ERK1/2, JNK and CREB phosphorylation as well as GRP78 and CHOP expression

**Figure 4:**
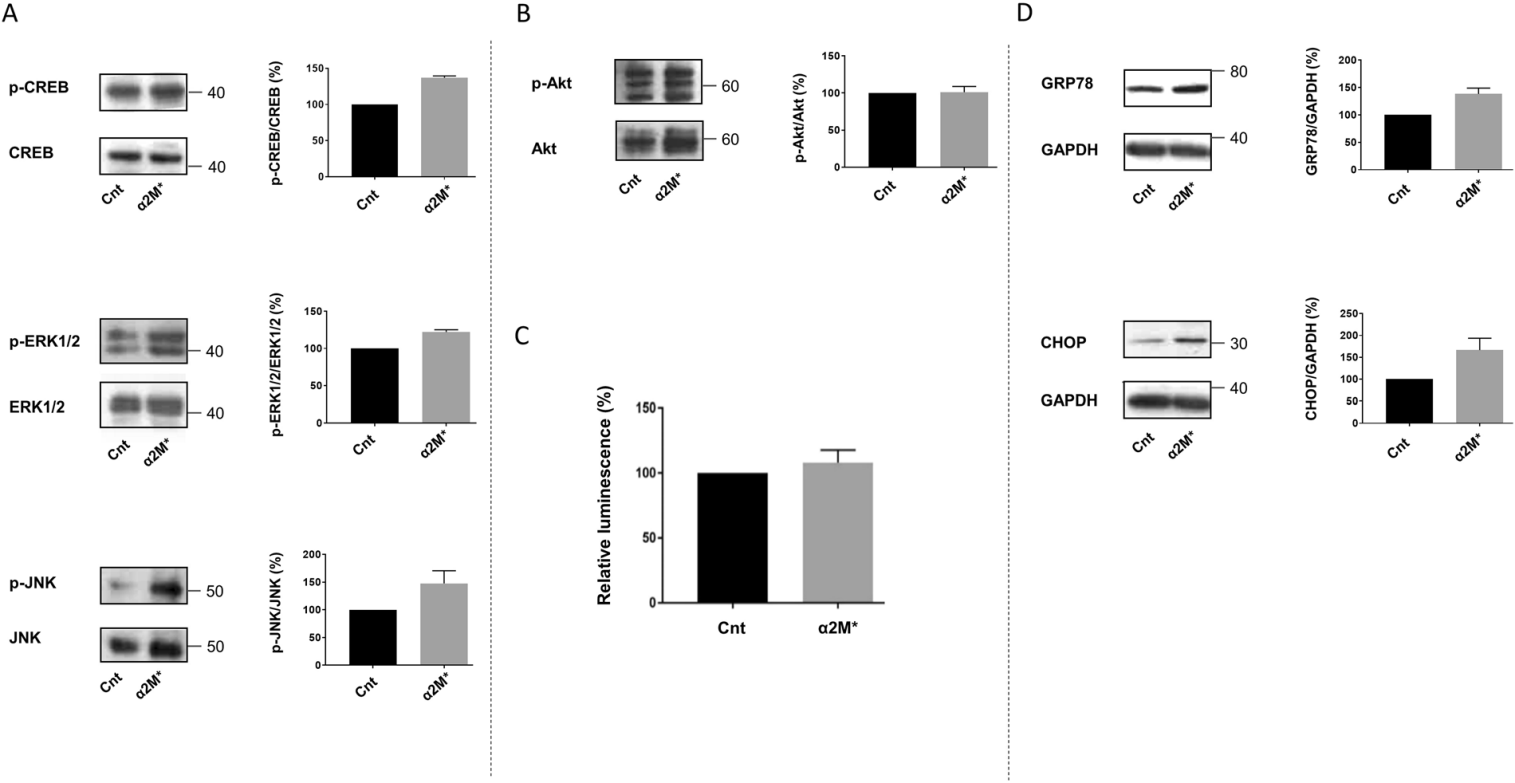
α_2_M* increases the expression of p-CREB, p-ERK1/2, p-JNK, p-Akt, GRP78 and CHOP. a-b. BeWo cells were seeded for 24 h prior to 24 h of starvation. Subsequently, cells were treated with or without 100 pM of α_2_M* for 30 min. Western blotting was performed. (**A**) p-CREB, CREB, p-ERK1/2, ERK1/2, p-JNK and JNK levels were quantified using the ImageJ software, and data are expressed as the fold change relative to the control. n = 3. (**B**) p**-**Akt and Akt levels were quantified using the ImageJ software, and data are expressed as the fold change relative to the control. n = 3. (**C**) BeWo cells transfected with a luciferase-expressing plasmid controlled by the NF-kB response element or a control plasmid were seeded and treated with or without 100 pM α_2_M*. 24 hours after treatment, the cells were lysed, and luciferase expression was measured with a Dual-Glo Luciferase assay system. *n* = 3. Data represented as mean±SEM, ns (not significant); t-test comparison test. (**D**) BeWo cells were seeded for 24 h prior to treatment with or without 100 pM of α_2_M* for 48 h. Western blotting was performed. GRP78 and CHOP levels were quantified using the ImageJ software, and data are expressed as the fold change relative to the control. n = 3. The images of bands for the target protein and GAPDH were taken from the same gel, and each image was cropped, as delineated by black dividing lines, as well as adjusted for image intensity for optimal visualisation.

The interaction of α_2_M* and cell membrane-located GRP78 activates several signalling pathways implicated in cell survival, growth and proliferation in prostate cancer, which have been reported to promote syncytialisation in trophoblastic cells (Fig. 1). The activation of these pathways was thus investigated in BeWo cells. Firstly, the protein levels of the active phosphorylated forms of several proteins — the expression of which was incremented in prostate cancer, concretely CREB, ERK1/2 and JNK —, were measured in BeWo cells (Fig. 4A) treated or not with α_2_M*. We observed increased expression of the phosphorylated forms of CREB, ERK1/2 and JNK upon α_2_M* treatment, suggesting that these proteins and their signalling pathways play a role in α_2_M*-cell fusion promotion. Subsequently, we decided to measure the expression of the phosphorylated form of Akt, the involvement of which in trophoblastic cell fusion is controversial. We detected no modulation of Akt phosphorylation after α_2_M* treatment in BeWo cells (Fig. 4B). Additionally, we tested the possible implications of NF-kB signalling in trophoblastic cell fusion by transfecting BeWo cells with a plasmid expressing the luciferase protein under NF-kB control. The luciferase glowing signal was not statistically different in cells treated or not with α_2_M* (Fig. 4C). These results suggest that Akt phosphorylation and NF-kB activity are not induced by α_2_M* in BeWo cells. Finally, we evaluated the expression of GRP78 and CHOP, the main markers of UPR activation (Fig. 4D), which is known to occur due to GRP78-α_2_M* interaction in prostate cancer cells. We observed an increased expression of CHOP and GRP78 upon α_2_M* treatment, suggesting the activation of UPR and its possible involvement in α_2_M*-dependent cell fusion promotion.

### α2M*-GRP78 interaction triggers several signalling pathways that are implicated in cell fusion

**Figure 5:**
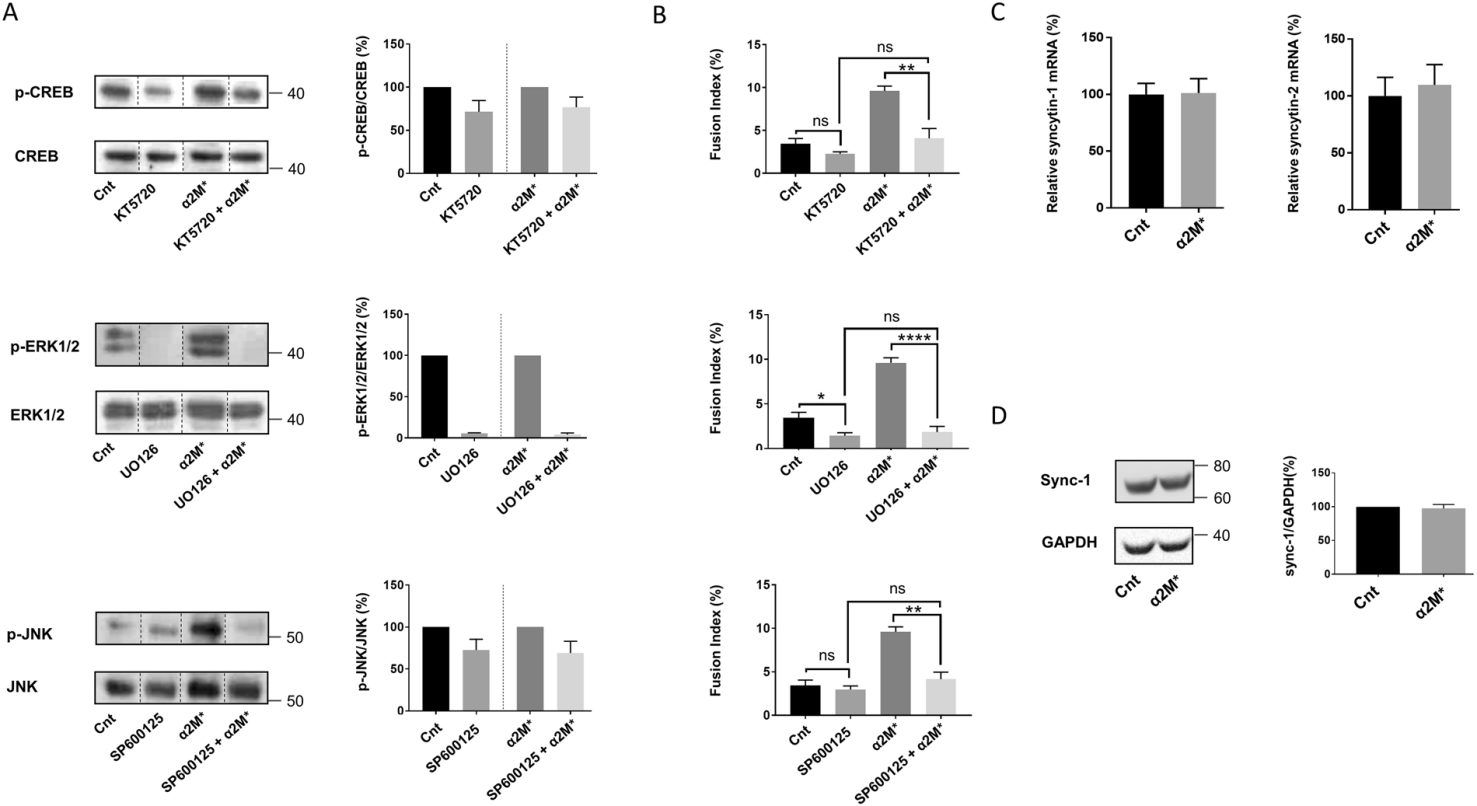
α_2_M* induced BeWo cell fusion through p-CREB, p-ERK1/2 and p-JNK activation, without affecting syncytin expression. (**A,B**) BeWo cells were seeded for 24 h prior to 24 h of starvation. Subsequently, cells were treated with 5 μM KT5720, 10 μM SP600125 or 10 μM UO126 for 1 h, and 100 pM of α_2_M* was added or not for 30 min. (**A**) Western blotting was performed. p-CREB, CREB, p-ERK1/2, ERK1/2, p-JNK and JNK levels were quantified using the ImageJ software, and data are expressed as the fold change relative to the control. n = 3. The images of bands for the target proteins were taken from the same gel, and each image was cropped, as delineated by black dividing lines, as well as adjusted for image intensity for optimal visualisation. (**B**) Nuclei and syncytia were counted, and a fusion index was calculated. *n* = 3. Data represented as mean±SEM. ns (not significant), *P ≤ 0.05, **P ≤ 0.01, ***P ≤ 0.005; ANOVA comparison test. (**C**,**D**) BeWo cells were seeded for 24 h prior to treatment with or without 100 pM of α_2_M*. (**C**) RNA was retrotranscribed, and 10 ng of cDNA was used to perform qPCR using syncytin-1 and syncytin-2 primers. *n* = 3. Data represented as mean±SEM. **P ≤ 0.01; t-test comparison test. (**D**) BeWo cells were seeded for 24 h prior to treatment with or without 100 pM of α_2_M* for 48 h. Western blotting was performed. Syncytin-1 and GAPDH levels were quantified using the ImageJ software, and data are expressed as the fold change relative to the control. n = 3. The images of bands for the target proteins were taken from the same gel, and each image was cropped, as delineated by black dividing lines, as well as adjusted for image intensity for optimal visualisation.

In order to elucidate the role of the different activated signalling pathways in trophoblastic cell fusion, selective inhibitors of the different activated proteins such as KT5720 (which inhibits PKA and thus affects CREB activation), UO126 (ERK1/2) and SP600125 (JNK) were used in BeWo cells. The expression of the different targeted proteins was measured by Western blot, observing a diminution in the expression of the phosphorylated active forms of CREB, ERK1/2 and JNK after treatment with the corresponding inhibitors (Fig. 5A). The phosphorylation reduction achieved by the inhibitors, in BeWo cells treated or untreated with α_2_M*, demonstrated the correct inhibition of the signalling pathways. Afterwards, the cell fusion rate was measured and compared with the corresponding controls before and after α_2_M* treatment (Fig. 5B). We then verified that these treatments did not alter cell viability that could affect the fusion index results (Fig. 1SA). CREB, ERK1/2 and JNK activate signalling pathways that are known to increase hCG secretion and the expression of syncytins in trophoblastic cells. We have already demonstrated that α_2_M* treatment does not affect hCG secretion (Fig. 3B). Therefore, we next investigated syncytin-1 and -2 expression in BeWo cells treated or not with α_2_M*. We observed that α_2_M* treatment did not affect their expression at the mRNA level (Fig. 5C). This result was also confirmed at the protein level for syncytin-1 (Fig. 5D), suggesting α_2_M*-GRP78 interaction played a role in cell fusion independent of hCG secretion and syncytin expression.

In order to elucidate the role of the different activated signalling pathways in trophoblastic cell fusion, selective inhibitors of the different activated proteins such as KT5720 (which inhibits PKA and thus affects CREB activation), UO126 (ERK1/2) and SP600125 (JNK) were used in BeWo cells. The expression of the different targeted proteins was measured by Western blot, observing a diminution in the expression of the phosphorylated active forms of CREB, ERK1/2 and JNK after treatment with the corresponding inhibitors (Fig. 5A). The phosphorylation reduction achieved by the inhibitors, in BeWo cells treated or untreated with α_2_M*, demonstrated the correct inhibition of the signalling pathways. Afterwards, the cell fusion rate was measured and compared with the corresponding controls before and after α_2_M* treatment (Fig. 5B). We then verified that these treatments did not alter cell viability that could affect the fusion index results (Fig. 1SA). CREB, ERK1/2 and JNK activate signalling pathways that are known to increase hCG secretion and the expression of syncytins in trophoblastic cells. We have already demonstrated that α_2_M* treatment does not affect hCG secretion (Fig. 3B). Therefore, we next investigated syncytin-1 and -2 expression in BeWo cells treated or not with α_2_M*. We observed that α_2_M* treatment did not affect their expression at the mRNA level (Fig. 5C). This result was also confirmed at the protein level for syncytin-1 (Fig. 5D), suggesting α_2_M*-GRP78 interaction played a role in cell fusion independent of hCG secretion and syncytin expression.

In order to elucidate the role of the different activated signalling pathways in trophoblastic cell fusion, selective inhibitors of the different activated proteins such as KT5720 (which inhibits PKA and thus affects CREB activation), UO126 (ERK1/2) and SP600125 (JNK) were used in BeWo cells. The expression of the different targeted proteins was measured by Western blot, observing a diminution in the expression of the phosphorylated active forms of CREB, ERK1/2 and JNK after treatment with the corresponding inhibitors (Fig. 5A). The phosphorylation reduction achieved by the inhibitors, in BeWo cells treated or untreated with α_2_M*, demonstrated the correct inhibition of the signalling pathways. Afterwards, the cell fusion rate was measured and compared with the corresponding controls before and after α_2_M* treatment (Fig. 5B). We then verified that these treatments did not alter cell viability that could affect the fusion index results (Fig. 1SA). CREB, ERK1/2 and JNK activate signalling pathways that are known to increase hCG secretion and the expression of syncytins in trophoblastic cells. We have already demonstrated that α_2_M* treatment does not affect hCG secretion (Fig. 3B). Therefore, we next investigated syncytin-1 and -2 expression in BeWo cells treated or not with α_2_M*. We observed that α_2_M* treatment did not affect their expression at the mRNA level (Fig. 5C). This result was also confirmed at the protein level for syncytin-1 (Fig. 5D), suggesting α_2_M*-GRP78 interaction played a role in cell fusion independent of hCG secretion and syncytin expression.

### CREB and ERK1/2 are involved in the control of UPR, which is implicated in cell fusion

**Figure 6:**
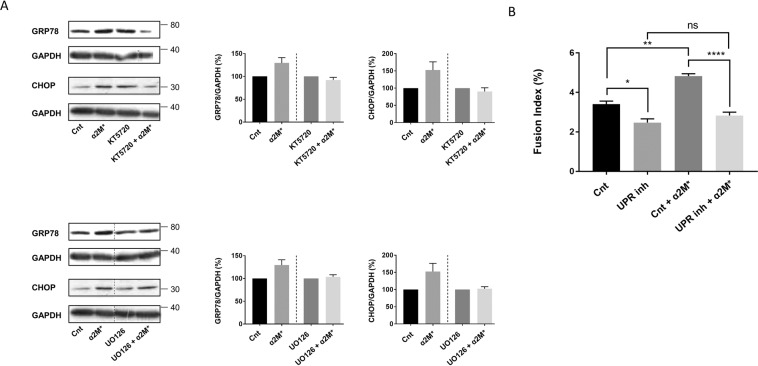
α_2_M*-induced p-CREB and p-ERK1/2 activation controls UPR triggering, which, in turn, modulates BeWo cell fusion. (**A**) BeWo cells were seeded for 24 h prior to treatment with or without 100 pM of α_2_M*, and 5 μM KT5720 or 10 μM UO126 for 48 h. Western blotting was performed. GRP78, CHOP and GAPDH levels were quantified using the ImageJ software, and data are expressed as the fold change relative to the control. n = 3. The images of bands for the target protein and GAPDH were taken from the same gel, and each image was cropped, as delineated by black dividing lines, as well as adjusted for image intensity for optimal visualisation. (**B**) BeWo cells were seeded for 24 h prior to treatment with or without 100 pM of α_2_M*, and 200 µM 4-(2-aminoethyl)benzenesulfonyl fluoride hydrochloride (AEBSF), 100 µM STF-083010 (STF) and 100 nM GSK2656157 (GSK) for 48 h. Nuclei and syncytia were counted, and a fusion index was calculated. *n* = 3. Data represented as mean±SEM. ns (not significant), *P ≤ 0.05, **P ≤ 0.01, ***P ≤ 0.005; ANOVA comparison test.

We decided to explore the possible modulation of UPR by CREB activation in trophoblastic cells since previous studies conferred this function to CREB^33^. Additionally, ERK1/2 is known to be an activator of CREB^34^, and we speculated that it could be involved in α_2_M-induced CREB phosphorylation and, consequently, UPR modulation. Firstly, we treated BeWo cells with KT5720, a potent PKA inhibitor preventing CREB phosphorylation (Fig. 6A), or U0216, a potent inhibitor of MKK1/2 preventing both ERK1/2 (Fig. 6A) and CREB phosphorylation (Fig. 2S). We then analysed their impact on the expression of UPR-related proteins, which we have already demonstrated to be involved in trophoblastic cell fusion promotion^16^. As we previously showed in Fig. 4A, we observed that the addition of α_2_M* to the BeWo cells without blocking CREB and ERK1/2 activation led to increased expression of GRP78 and CHOP (Fig. 6A). On the contrary, BeWo cells that were pre-treated with KT5720 or UO126 did not show increased expression of GRP78 or CHOP after the addition of α_2_M*. These results suggest that α_2_M*-induced CREB and ERK1/2 activation may affect BeWo cell fusion through UPR activation. Finally, we blocked UPR activation by pre-treating BeWo cells before α_2_M* addition with inhibitors of the different UPR branches (4-(2-aminoethyl) benzenesulfonyl fluoride hydrochloride (AEBSF), STF-083010 (STF) and GSK2656157 (GSK)). α_2_M*-induced cell fusion is inhibited by inhibitors of UPR (Fig. 6B), while not affecting cell viability (Fig. 1SB), suggesting that UPR mediates the effect of α_2_M* on BeWo cell fusion.

## Discussion

PE and other pregnancy disorders are characterised by incorrect STB formation [review^35^] and, therefore, abnormal placentation, together with reduced expression of cell membrane-located GRP78 in trophoblastic PE cells^19^. Syncytialisation seems to be essential for correct placental development, and understanding the mechanisms controlling this process is fundamental to medical treatment. α_2_M* bound to cell surface-located GRP78 has been found to activate some signalling pathways in cancer cells, leading to cell survival, growth and proliferation^22,29^. Some of the proteins implicated in these signalling pathways have been reported to induce syncytin expression and hCG secretion, inducing cell fusion^30^. We hypothesised that some of these signalling pathways could also be activated by α_2_M*-GRP78 interaction in trophoblastic cells and, therefore, be implicated in cell fusion.

**Figure 7:**
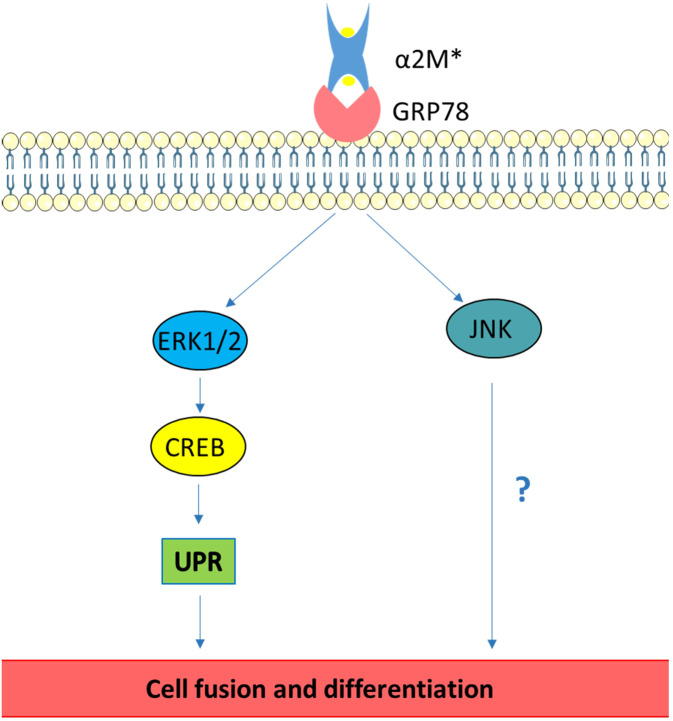
Schematic representation of signalling cascades involved in trophoblastic cell fusion stimulated by α2M*-GRP78 interaction. Interaction between GRP78 and α2M* promotes the activation of ERK1/2 and CREB, which, in turn, enhances UPR activation, inducing cell fusion in BeWo cells. Additionally, GRP78 interaction with α2M* enhances JNK activation, increasing BeWo cell fusion by an unknown mechanism.

In this study, we have taken advantage of BeWo cells, a cell line that is used as a trophoblastic cell fusion model, which lacks α_2_M expression. The characteristics of this cell line allowed us to study the effect of exogenous α_2_M in trophoblastic cells and the involvement of GRP78 during the different triggered events. Firstly, we demonstrated that α_2_M* treatment led to an increase in cell fusion through GRP78 interaction, phosphorylation of ERK1/2, CREB and JNK, and UPR activation. The ways these proteins promote cell fusion have not been completely elucidated; we evaluated syncytin-1 and -2 expression and hCG secretion since they were previously demonstrated to be increased by ERK1/2, CREB and/or JNK activation^30,31^. However, we obtained negative results, showing α_2_M*-GRP78 interaction affects trophoblastic cell fusion independent of hCG secretion and syncytin expression. This unexpected result led us to hypothesise that CREB and ERK1/2 could be implicated in trophoblastic cell fusion by different mechanisms. It was reported that CREB phosphorylation had an impact on UPR activation in breast cancer cells^33^. Furthermore, we recently demonstrated that UPR activation in trophoblastic cells is involved in syncytialisation^16^. In this paper, we have demonstrated that α_2_M* treatment of BeWo cells induced UPR activation, and, thus, cell fusion but not differentiation. We have also shown that inhibition of CREB phosphorylation (using PKA or ERK1/2 inhibitors) eliminated the effect of α2M* on UPR activation in BeWo cells, suggesting that α_2_M* could activate the CREB pathway and, thus, UPR in BeWo cells (Fig. 7).

However, the impact of α_2_M*-induced CREB and ERK1/2 activation on UPR activation and, consequently, on trophoblastic syncytialisation was surprising since we previously demonstrated that UPR was implicated in both cell fusion and differentiation (hCG secretion)^16^, while, in this study, we have only observed effects on cell fusion. Conveniently, it was observed that CREB modulates the IRE1α and PERK branches of the UPR^33^, while no effect was described in the ATF6 branch. We have seen that UPR-induced hCG secretion is controlled primarily by ATF6 (data not shown) in trophoblastic cells. This observation may explain why α_2_M*-induced UPR activation does not lead to increased hCG secretion.

Interestingly, we also observed an increased trophoblastic expression of α_2_M with gestational age, displaying strong α_2_M expression at term, which suggests that this glycoprotein plays a role in pregnancy. Some previous observations already highlighted the implication of α_2_M in pregnancy by using murine models^36,37^. It was demonstrated that α_2_M participates in the spiral artery remodelling, an essential step necessary for the successful pouring of blood into the placenta^36^. Additionally, it was proven that murine α_2_M and other members of the α_2_M family, namely the murinoglobulins, were implicated in trophoblastic invasion^37^. Moreover, functional deficiency of α_2_M has not been described, suggesting that α_2_M is essential for gestational success^37^. Nevertheless, the role of human α_2_M in spiral artery remodelling was never studied. Here, for the first time, we suggested that α_2_M* could also play an essential role in trophoblastic cell fusion. Unfortunately, we were unable to abolish or significantly decrease α_2_M expression in primary cells to confirm the role of endogenous α_2_M in the fusion of trophoblastic cells, and we had to pursue our investigation by using BeWo cells. Nevertheless, we managed to unravel a new mechanism by which α_2_M is implicated in pregnancy, demonstrating the importance of this protein to gestation.

The results obtained in this study regarding the impact of α_2_M* on trophoblastic cell fusion lead us to hypothesise that members of the same family, such as the pregnancy zone protein (PZP), could have similar effects. PZP is a macroglobulin protein, normally detected as a trace plasma protein (<0.01 mg/ml), the expression of which is highly modulated by reproductive hormones, detectable by week 5 of pregnancy and peaking at term^38^. Blood concentrations at term have been reported to be 100-200-fold above pre-conception levels and matches, or even exceeds, that of α_2_M^39^. This pregnancy protein shares a 71% homology with α_2_M^40^, and it has been identified in syncytiotrophoblast, immune cells, placental villous mesenchyme and endothelium^41^. The PZP functions have been hypothesised to be similar to those attributed to α_2_M^42,43^. Additionally, it was reported that PZP possesses a receptor-binding domain with high homology to the one observed in α_2_M^28,43^. We speculate that due to its elevated expression during pregnancy and its high homology to α_2_M^38^, PZP may bind GRP78 and also play a role in trophoblastic cell fusion by activating similar signalling pathways to those activated by α_2_M*. Unfortunately, this possibility has not been investigated yet, and it could be essential to understanding the potential mechanisms triggered by PZP during pregnancy.

Furthermore, looking from a structural perspective, it is evident that macroglobulin proteins are oligomeric complexes formed by several identical subunits. α_2_M is a homo-tetrameric protein, and, therefore, it contains four different receptor-binding domains, one per monomer; while PZP is a homo-dimer, containing two receptor-binding domains^28,38^. This spatial conformation could provide α_2_M and PZP with the ability to bind to several GRP78 proteins from different cells, conferring to these proteins a *bridge* function, bringing cells together in close proximity and favouring cell fusion. It may be interesting to investigate whether the monomeric versions of α_2_M could induce trophoblastic cell fusion or if the *bridge* function derived from the structural conformation of this protein favours cellular interactions and is needed for the attainment of total fusion competence.

We could conclude that the cell surface-located GRP78 is implicated in trophoblastic cell fusion through the interaction of α_2_M* and the subsequent activation of ERK1/2 and CREB, which, in turn, modulates UPR activation in BeWo cells. These results reinforce the critical role of GRP78 and UPR in trophoblastic cell fusion^16,19^ and encourage further investigation into the roles of α2M family proteins during pregnancy.

## Materials and Methods

### Ethics statement

This research was approved by the Geneva Hospital Ethics Committee (#10-001 and 02-088). Informed written consent was obtained from all patients before inclusion in the study. All methods were carried out in accordance with relevant guidelines and regulations.

### Purification of vCTB

vCTB were isolated from first-trimester trophoblast (n = 3 early first trimester, n = 3 late first trimester) and normotensive term placentae (n = 3). Purification took place according to the protocol previously detailed by Bischof *et al*.^44^ Briefly, isolation of small placental tissue pieces was followed by enzymatic tissue digestion with a Difco Trypsin solution (BD, Le Pont de Claix, France). Next, cell separation was performed in a Percoll gradient (GE Healthcare, Uppsala, Sweden), and immunopurification of the vCTB was performed using monoclonal mouse anti-human CD45 immobilised antibodies (Dako, Glostrup, Denmark).

### Cell culture

BeWo cells (ATCC, CCL-98, Molsheim, France) were kindly furnished by Dr Thierry Fournier (INSERM U767, Paris, France) and cultured at 37 °C and 5% CO2 in Ham’s F12K medium (Gibco, Invitrogen, Basel, Switzerland), supplemented with 0.05 mg/ml gentamycin (Invitrogen, Basel, Switzerland) and 10% FBS (Biochrom AG, Oxoid AG, Basel, Switzerland).

vCTB purified from placenta were cultured in Dulbecco’s modified Eagle’s medium (DMEM; Gibco, Invitrogen, Basel, Switzerland), supplemented with 0.05 mg/ml gentamycin and 10% FBS under the same conditions.

### Cell treatments

To evaluate the fusion capacity of BeWo cells under different conditions, BeWo cells were treated 24 h post-seeding for 48 h with or without 20 µM Forskolin (Sigma, St Louis, MO, USA) to induce syncytialisation and 100 pM of α_2_M* purified and activated as previously described^45^ (three independent experiments). Briefly, insoluble material from human plasma was pelleted, and the supernatant plasma solution was dialyzed prior to performing metal chelate chromatography in a zinc-sepharose-4B column. Bound protein was pulsed from the column, and peak protein fractions were pooled and concentrated prior to gel filtration. The high molecular weight peak containing pure α2M was pooled and concentrated for storage.

To evaluate the role of membrane GRP78 in α_2_M*-induced cell fusion, a pre-treatment with rabbit anti-GRP78 antibodies (GL-19, 3 µg/ml from Sigma, Darmstadt, Germany) or normal rabbit IgG antibodies (sc-2027, 3 µg/mL from SantaCruz Biotechnology, Labforce, Switzerland) was performed in a 96-well tissue culture plate (Falcon, Durham, NC, USA) 24 h post-seeding. Simultaneously, 20 µM Forskolin (Sigma, St Louis, MO, USA) was added to the different wells to induce syncytialisation, and 6 h later, 100 pM of α_2_M* was added and left for 48 h (three independent experiments).

To evaluate the role of UPR activation in α_2_M*-induced cell fusion, BeWo cells were treated with different UPR inhibitors: 100 nM GSK2656157 (Selleckchem, Zurich, Switzerland), 200 µM AEBSF (Sigma, Darmstadt, Germany) and 100 µM STF-083010 (Selleckchem, Zurich, Switzerland). 6 h later, cells were also treated or not with 100 pM of α_2_M* for 48 h (three independent experiments).

To evaluate the role of ERK1/2, JNK, and CREB phosphorylation in α_2_M*-induced cell fusion, cells were treated with 5 μM KT5720 (Sigma, Darmstadt, Germany), 10 μM SP600125 (Sigma, Darmstadt, Germany) or 10 μM UO126 (Sigma, Darmstadt, Germany) for 1 h after 24 h of starvation. Afterwards, 100 pM of α_2_M* was added to the cells for 30 min (three independent experiments).

To evaluate the role of ERK1/2, JNK and CREB phosphorylation in the UPR through α_2_M*, cells were treated with 5 μM KT5720, 10 μM SP600125 or 10 μM UO126. At the same time, 100 pM of α_2_M* was added to the cells for 48 h (three independent experiments).

Cell fusion index, luciferase expression and cell viability assays were conducted in 96-well plates (Falcon, Durham, NC, USA) after the seeding of 15,000 cells/well.

The experiments performed in 6-well plates (Falcon, Durham, NC, USA), Western Blot, qPCR and β-hCG measurement, were done after the seeding of 300,000 cells/well.

### Cell transfection

BeWo cells were transfected 24 h post-seeding with a NF-kB luciferase reporter vector that contained a firefly luciferase gene under the control of a multimerised NF-kB-responsive element located upstream of a minimal promoter (pGL4.32[luc2P/NF-kB/ Hygro], E8491, Promega Madison, WI, USA) or a control vector (a non-inducible luciferase vector that contains a firefly luciferase gene under the control of a minimal promoter, without any additional response elements; pGL4.15[luc2P/Hygro], E6701, Promega Madison, WI, USA) (three independent experiments). JetPei transfection reagent was used to transfect cells following the manufacturer’s instructions (Polyplus transfection, Illkirch, France). The experiments were performed in a 6-well tissue culture plate (Falcon, Durham, NC, USA) using 3 µg of the different vectors. BeWo cells were treated 24 h after transfection with 100 pM of α_2_M* for 24 h.

The control vector was used to determine the NF-kB pathway-specific effects and background luciferase activity (ratio of firefly luminescence from the NF-kB reporter to firefly luminescence from the control firefly luciferase vector).

### Polymerase chain reaction (PCR)

The extraction of total RNA was performed in BeWo cells (from three independent experiments) or vCTB (n = 3 for each gestational stage) using the PureLinkRNA Mini Kit (AMBION, Austin, TX, USA). Reverse transcription with 1 µg of total RNA was completed using the High Capacity cDNA Reverse Transcription Kit (Applied Biosystems, Life Technologies). A PCR using 50 ng of cDNA was performed using the REDTaq ReadyMix PCR Reaction Mix (Sigma, Steinheim, Germany) and the following primers for α_2_M detection: α_2_M forward 5′-GAAGTGTTTGGGACCAGATG-3′ and α_2_M reverse 5′-AGTCGGAAGCGTCACTATAC-3′; and GAPDH detection: GAPDH forward 5′-CGTATTGGGCGCCTGGTCACC-3′ and GAPDH reverse 5′-GGGATGATGTTCTGGAGAGCCC-3′. A 1% agarose gel was prepared using Basic agarose premier (MP Biomedicals, Illkirch, France) and SYBR Safe DNA gel stain (Cartshad, CA, USA), as indicated by the manufacturer. Acquisition was accomplished using the Geneflash machine (Syngene Bioimaging).

### Quantitative polymerase chain reaction (qPCR)

PureLinkRNA Mini Kit (AMBION, Austin, TX, USA) was used to extract total RNA from BeWo (from three independent experiments). Reverse transcription was performed with 1 µg of total RNA using the High Capacity cDNA Reverse Transcription Kit (Applied Biosystems, Life Technologies). Detection of the real-time qPCR product was performed using the KAPA SYBR FAST qPCR Kit Master Mix (Kapa Biosystems, Axon Lab, Baden, Switzerland) on an Eco Real-Time PCR System (Labgene Scientific, Châtel-St-Denis, Switzerland). The relative expression of syncytin-1 and syncytin-2 genes was normalised to the two housekeeping genes GAPDH and Cyclophilin A. The primers used for quantification of these genes’ expression are described in Table 1.

**Table 1:** List of qPCR primers used in the study.

Gene name	Forward sequence	Reverse sequence
Syncytin 1	5′-CCCAGGCGTTAGGTATACGA-3′	5′-GACCTTCCCTGAGGACTGTG-3′
Syncytin 2	5′-CCTTCACTAGCAGCCTACCG-3′	5′-GCTGTCCCTGGTGTTTCAGT-3′
GAPDH	5′-CGACCACTTTGTCAAGCTCA-3′	5′-CCCTGTTGCTGTAGCCAAAT-3′
Cyclophilin A	5′-TACGGGTCCTGGCATCTTGT-3′	5′-CCATTTGTGTTGGGTCCAGC-3′

### Western blot

As previously described by Bastida-Ruiz *et al*.^16^, 40 µg of proteins from whole BeWo (from three independent experiments) was fractionated by sodium dodecyl sulfate-polyacrylamide gel electrophoresis and transferred to nitrocellulose membrane. The antibodies used for immunoblotting are described in Table 2. A specific signal was detected using Amersham ECL Prime Western Blotting Detection Reagent (GE Healthcare, Buckinghamshire, UK) or Immobilon Western Chemiluminescent HPR Substrate (Billerica, MA, USA).

**Table 2:** The list of antibodies used in the study.

Antibody	Reference	Company	Dilution	Species
anti-desmoplakin	sc-390975	Santa Cruz Biotechnology	1 :500	mouse
anti-ERK1/2	9102 S	Cell Signaling	1:1,000	Rabbit
anti-phospho-ERK1/2	4376 S	Cell Signaling	1:1,000	rabbit
anti-JNK antibodies	9252 S	Cell Signaling	1:1,000	rabbit
anti-phospho-JNK	9251 S	Cell Signaling	1:1,000	rabbit
anti-CREB	sc-186	Santa Cruz Biotechnology	1:500	rabbit
anti-phospho-CREB	sc-7978	Santa Cruz Biotechnology	1:500	goat
anti-Akt	sc-8312	Santa Cruz Biotechnology	1:500	rabbit
anti-phospho-Akt	9271 S	Cell Signaling	1:1,000	rabbit
anti-GRP78	GL-19	Sigma	1:3,000	rabbit
anti-CHOP	sc-7351	Santa Cruz Biotechnology	1:500	mouse
anti-syncytin-1	sc-30640	Santa Cruz Biotechnology	1:500	goat
Anti-GAPDH	MAB374	Millipore	1:10,000	mouse
anti-mouse IgG-HRP	sc-2005	Santa Cruz Biotechnology	1:3,000	goat
anti-goat IgG-HRP	sc-2354	Santa Cruz Biotechnology	1:3,000	mouse
anti-rabbit IgG (H + L)-HRP	170–6515	Bio-Rad	1:3,000	goat

### Fusion index (FI)

A frequently used marker of trophoblastic cell fusion is the FI^46^. It describes the number of nuclei inside syncytia as a percentage of the total number of nuclei.

Trophoblastic cell FI was determined by immunocytochemistry, as previously described by Bastida-Ruiz *et al*.^16^. Briefly, cells were washed in PBS, fixed in 3% paraformaldehyde and immunostained using mouse anti-desmoplakin antibodies (sc-390975, 1:500 dilution from Santa Cruz Biotechnology, Heidelberg, Germany). Revelation was performed with diaminobenzidine (Dako, Carpinteria, CA, USA) after incubation with secondary antibodies anti-mouse IgG-HRP (sc-2005, Santa Cruz Biotechnology, Heidelberg, Germany). Nuclei were stained with haematoxylin. Image acquisition was completed using a Ceti Inverso TC-100 inverted biological microscope (Medline scientific, UK). FI was expressed in percentages and calculated as follows: [(N − S)/T], where N equals the number of nuclei in syncytia, S equals the number of syncytia, and T equals the total number of nuclei counted^19^. The syncytia are recognised as cells containing several nuclei inside the same cell. Three different fields were analysed per well for each experiment. FI was calculated for at least three independent experiments, run in triplicate.

### β-hCG measurement

Cell culture medium from BeWo cells was collected and centrifuged at 14,000 × g for 5 min. The amount of β-hCG in the supernatant was measured by enzyme-linked immunosorbent assay (DRG International, Diagnostik Medizintechnik, Oberdof, Switzerland) according to the instructions provided by the manufacturer. Results were normalised by the total cellular protein content of corresponding wells. β-hCG measurement was done for three independent experiments, run in triplicate.

### Luciferase expression

BeWo cells were lysed, without discarding the supernatant, with the Dual-Glo Luciferase assay system (Promega, Madison, WI, USA) following the manufacturer’s instructions 24 hours after treatment. The resulting lysate was used to analyse the luminescence with the GloMax 96-well Luminometer (EG501, Promega Biosystems Sunnyvale, Inc., Sunnyvale, CA, USA) and the Glomax 1.9.3 software. Luciferase expression measurement was done for three independent experiments, run in triplicate.

### Proliferation/viability assay

To determine the effect of the different treatments on BeWo cell viability, we performed an MTT assay. After the corresponding treatment, the medium was replaced with a medium containing 20% MTT (Sigma-Aldrich Corporation, USA) solution (5 mg/mL in medium) for 2 h. Acidic isopropanol solution (150 µL) was added, and then each well was vigorously mixed to dissolve the precipitated formazan. UV–visible absorption was measured at 540 and 690 nm. A proliferation/viability assay was performed for three independent experiments, run in triplicate.

### Statistics

Data were represented as means ± standard error of the mean (SEM) for at least 3 different samples. Statistical differences between samples were assessed by the Student’s t test or ANOVA test, followed by Tukey’s multiple comparison test, as specified in each experiment, and the p-value<0.05 was considered significant. GraphPrism software was used to perform the different statistical analyses.

## Supplementary information

Supplemental information.
