# Supplementary material for: Activated α2-macroglobulin binding to cell surface GRP78 induces trophoblastic cell fusion
Source: Sci Rep. 2020 Jun 15;10:9666. doi: 10.1038/s41598-020-66554-0 (PMC7295802; doi:10.1038/s41598-020-66554-0)

**Supplementary Information**

**Activated α_2_-macroglobulin binding to cell surface GRP78 induces trophoblastic cell fusion**

Daniel Bastida-Ruiz^1^, Christine Wuillemin^1^, Aude Pederencino^1^, Michal Yaron^1^, Begoña Martinez de Tejada^1^, Salvatore Vincent Pizzo^2^, Marie Cohen^1^*.

1. Department of Pediatrics, Gynecology and Obstetrics, Faculty of Medicine, University of Geneva, 1206, Geneva, Switzerland.

2. Department of Pathology, Duke University Medical Center, Durham, North Carolina 27710, USA.

* corresponding author [marie.cohen@unige.ch](mailto:marie.cohen@unige.ch)

**Supplementary legends**

**Supplementary Figure 1. Effects of KT5720, SP600125, UO126 and 3 UPR inhibitors on BeWo cell viability. a.** BeWo cells were starved 24 h prior to treatment with 5 μM KT5720, 10 μM SP600125 or 10 μM UO126 for 1 h and subsequently, with or without 100 pM of α_2_M* for 30 min. Cell viability assay was performed. n=3. Data represented as mean ±SEM. ns (not significant); ANOVA comparison test. **b.** BeWo cells were seeded 24 h prior to treatment with or without 100 pM of α_2_M* and 200 µM 4-(2-aminoethyl)benzenesulfonyl fluoride hydrochloride (AEBSF), 100 µM STF-083010 (STF) and 100 nM GSK2656157 (GSK) for 48h. Cell viability assay was performed. n=3. Data represented as mean ±SEM. ns (not significant); ANOVA comparison test.

**Supplementary Figure 2. p-ERK1/2 modulates the phosphorylation of CREB**. BeWo cells were starved for 24 h prior to treatment with or without 10 μM UO126, followed or not by a treatment of 100 pM of α_2_M* for 30 min. Western blotting was performed. p-CREB and CREB levels were quantified using the ImageJ software, and data are expressed as the fold change relative to the control. n = 3. The images of bands for the target protein and GAPDH are taken from the same gel and each image has been cropped as delineated by black dividing lines as well as adjusted for image intensity for optimal visualization.

**Supplementary Figure 3. Full images of western blots shown in Figure 4-6 and Supplementary Figure 1. a.** From these western blot images were taken the p-CREB and CREB fragments used in Figure 4A (red dotted lines indicate the cropping locations), 5A (yellow dotted lines indicate the cropping locations) and S1 (blue dotted lines indicate the cropping locations). **b.** From these western blot images were taken the p-ERK1/2 and ERK1/2 fragments used in Figure 4A (red dotted lines indicate the cropping locations) and 5A (yellow dotted lines indicate the cropping locations). **c**. From these western blot images were taken the p-JNK and JNK fragments used in Figure 4A (red dotted lines indicate the cropping locations) and 5A (yellow dotted lines indicate the cropping locations). **d.** From these western blot images were taken the p-Akt and Akt fragments used in Figure 4B (red dotted lines indicate the cropping locations). **e.** From these western blot images were taken the GRP78 and corresponding GAPDH fragments used in Figure 4D (red dotted lines indicate the cropping locations) and 6A (purple dotted lines indicate the cropping locations). **f.** From these western blot images were taken the CHOP and corresponding GAPDH fragments used in Figure 4D (red dotted lines indicate the cropping locations) and 6A (purple dotted lines indicate the cropping locations). **g.** From these western blot images were taken the syncytin-1 and corresponding GAPDH fragments used in Figure 5D (green dotted lines indicate the cropping locations).

**Supplementary Figures**

**Supplementary Figure 1**

**
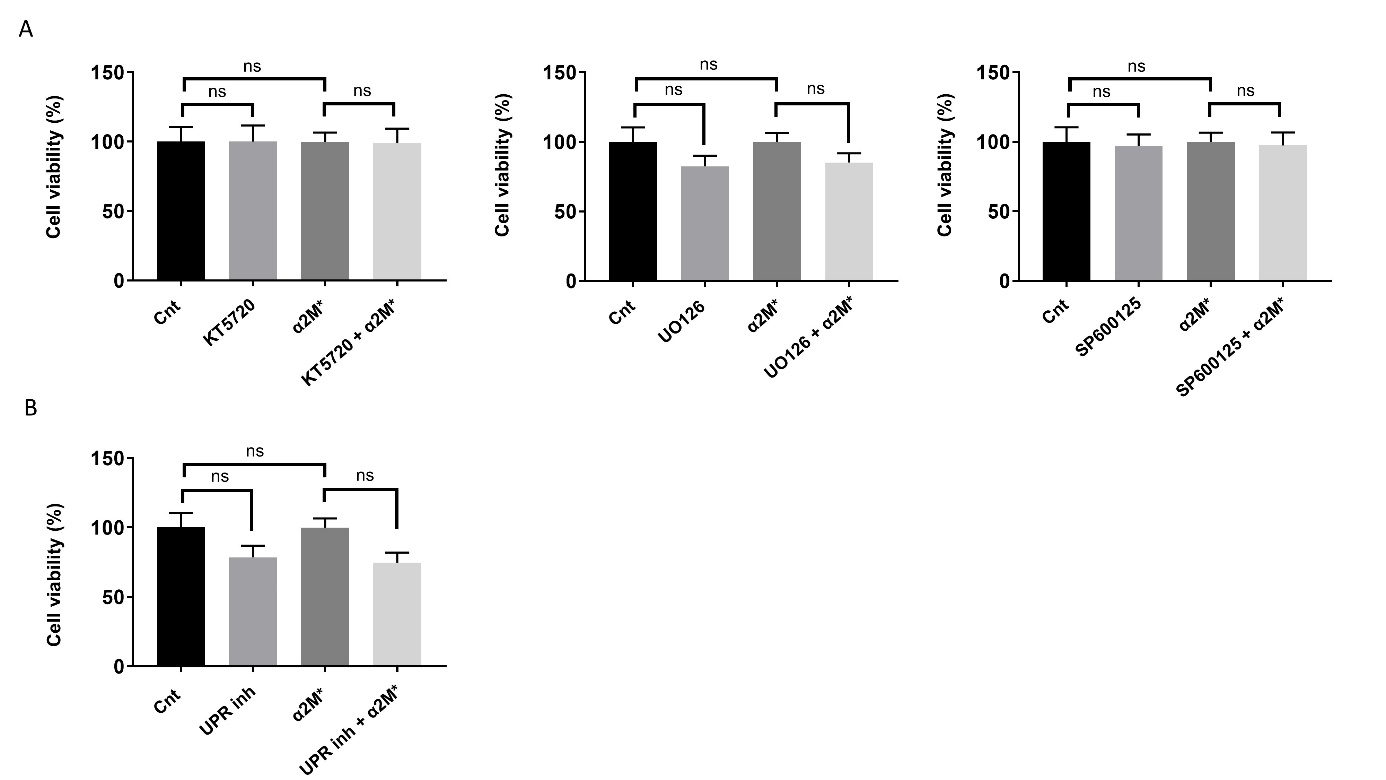
**

**Supplementary Figure 2**


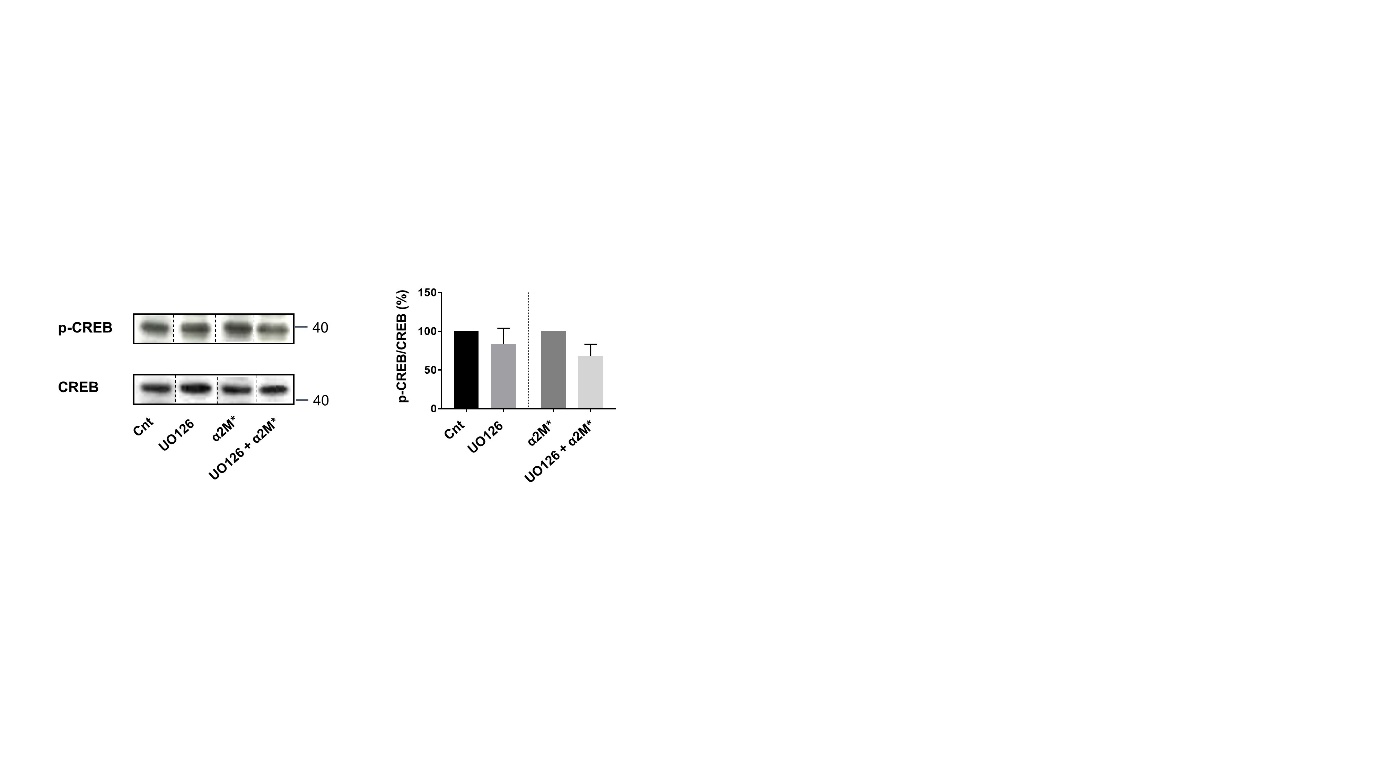


**Supplementary Figure 3**


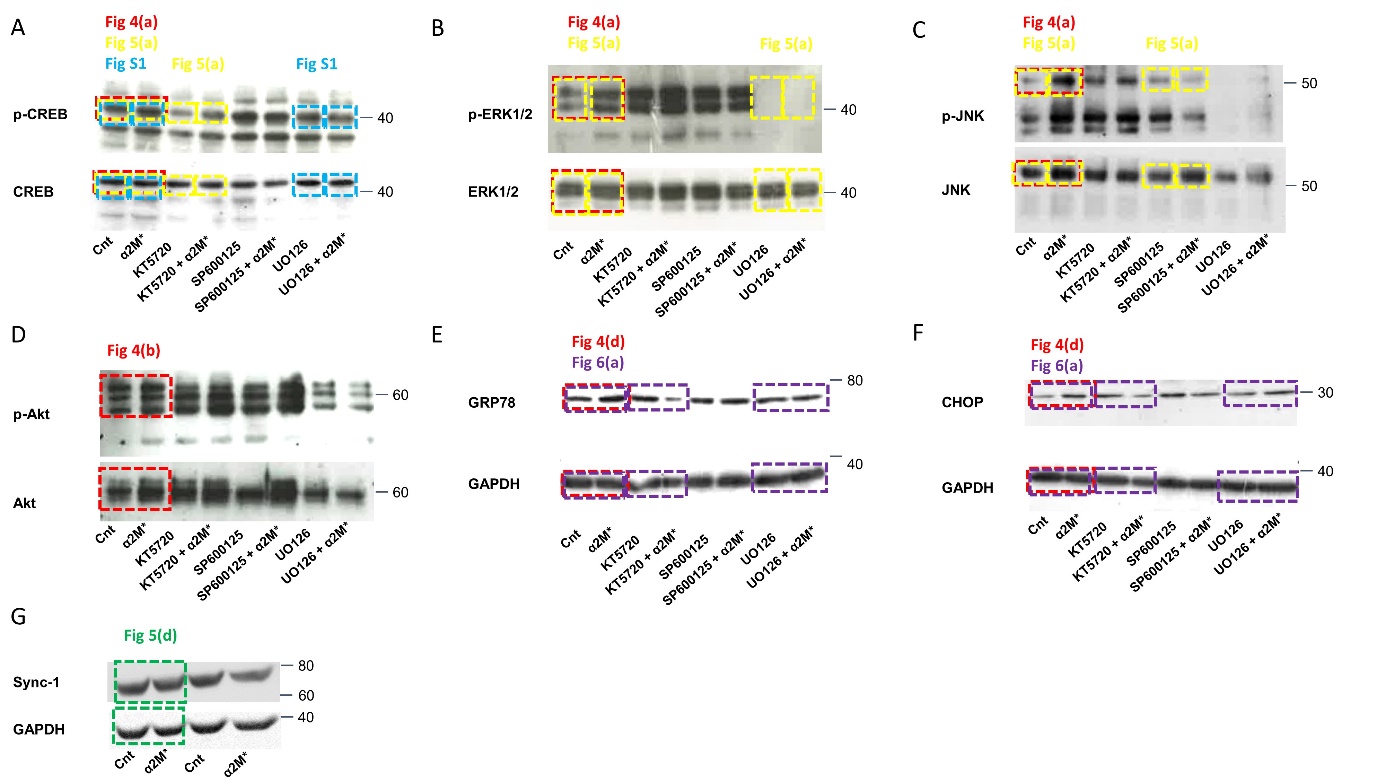

Supplement: Supplementary file 1 — Supplemental information. [file 41598_2020_66554_MOESM1_ESM.docx]
